# A novel mutation in *SETD1A* is associated with early-onset epilepsy—a rare case report

**DOI:** 10.3389/fnins.2026.1864983

**Published:** 2026-07-09

**Authors:** RiNa Su, Lei Zhu, Lin-Xia Jiang, Ya-Li Li, Liang-Liang Fan

**Affiliations:** 1 Department of Obstetrics and Gynecology, Ordos Clinical Medical College of Inner Mongolia Medical University, Ordos Central Hospital, Ordos, Inner Mongolia, China; 2 Department of Cell Biology, School of Life Sciences, Central South University, Changsha, China; 3 Department of Reproductive Genetics, Hebei General Hospital, Shijiazhuang, China

**Keywords:** epilepsy, H3K4 methyltransferase, mutation, neurodevelopmental disorders, SETD1A

## Abstract

SET Domain Containing 1A (SETD1A) is a histone H3K4 methyltransferase implicated in neurodevelopmental disorders. Pathogenic variants in this gene are associated with schizophrenia, intellectual disability, and epilepsy. Here, whole-exome sequencing and Sanger sequencing were performed on a 4-year-old Chinese girl with early-onset epilepsy and her unaffected parents. A novel *de novo* heterozygous variant in *SETD1A* (NM_014712.3: c.1067C > T/p.Ser356Phe) was identified in a patient presenting with focal-to-bilateral tonic–clonic seizures, beginning at 3 months of age. Neuroimaging revealed a normal brain MRI, and comprehensive neuropsychological assessment indicated preserved cognitive function. The variant was absent in 200 local controls and classified as likely pathogenic per ACMG criteria. This study may expand the mutation and phenotypic spectrum of SETD1A-related disorders, establishing the relationship between SETD1A variants and isolated early-onset epilepsy without accompanying severe neurodevelopmental deficits, highlighting the value of genetic testing in infants with unexplained epilepsy.

## Introduction

Epilepsy is a chronic neurological disorder defined by recurrent, transient episodes of abnormal neuronal activity, attributable to excessive and synchronous neuronal discharges in the brain (Pease et al., 2024; Elder et al., 2025). Clinically, it presents with heterogeneous manifestations, including impaired consciousness, motor convulsions, sensory disturbances, and other paroxysmal symptoms, and affects individuals across all age groups. Globally, the annual incidence ranges from 40 to 70 cases per 100,000 individuals (Nandan et al., 2023; Cacciapuoti et al., 2025). Early-onset epilepsy, particularly within the first 2 years of life, represents a common and clinically distinct phenotype of childhood neurological dysfunction, with an incidence of 70.1 per 100,000 (Hunter et al., 2020). Historically, numerous infantile epileptic syndromes were categorized as “idiopathic.” However, recent advances in genetic testing, particularly the widespread implementation of next-generation sequencing, have facilitated the systematic identification of underlying monogenic and polygenic etiologies (Dwivedi et al., 2024). Consequently, molecular genetics research in epilepsy has advanced rapidly over the past decade (Verma et al., 2021). Given the marked genetic heterogeneity across early-onset epilepsy subtypes, establishing a precise genetic diagnosis holds substantial clinical utility for prognostication, targeted therapeutic selection, and familial counseling.

SETD1A (SET Domain Containing 1A) is a pivotal histone lysine methyltransferase and a core member of the mammalian SET1 family of histone methyltransferases (Chen et al., 2023). The C-terminal region of the SETD1A protein harbors highly conserved SET and post-SET domains, which constitute its catalytic core and are indispensable for enzymatic activity (Alicea-Velazquez et al., 2016). As a dedicated histone H3 at lysine 4 (H3K4) specific methyltransferase, SETD1A mediates mono-, di-, and trimethylation of H3K4 in an S-adenosylmethionine dependent manner (Hoshii et al., 2024). Among these modifications, H3K4 trimethylation (H3K4me3) serves as a well-established epigenetic hallmark of transcriptionally active promoters and is critically involved in neurodevelopmental processes, including neuronal differentiation, synaptogenesis, and synaptic plasticity (Lewerissa et al., 2024). Emerging evidence indicates that pathogenic variants in the *SETD1A* gene are associated with early-onset epilepsy in pediatric populations (Yu et al., 2019).

Here, we recruited a 4-year-old girl present with epilepsy. By employing whole exome sequencing and Sanger sequencing, we detected a *de novo* mutation (NM_014712.3: c.1067C > T/p.S356F) of *SETD1A* gene in the patient.

### Case presentation

**Figure 1:**
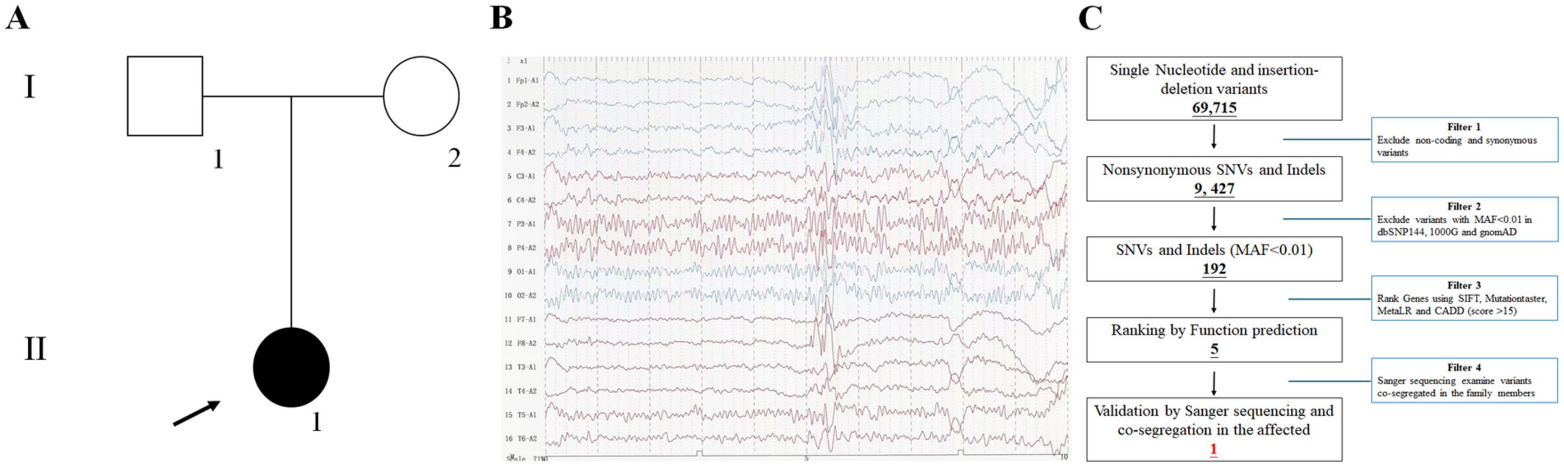
Clinical data of the family. **(A)** Pedigree of the family affected by early-onset epilepsy. Family members are identified by generations and numbers. Squares indicate male family members; circles, female members; Black closed symbols, the affected individual; open symbols, unaffected members; arrow, proband. **(B)** A representative scalp electroencephalogram (EEG) recorded during the wakeful resting state is presented, acquired using the standard international 10–20 electrode placement system with ipsilateral earlobe references (A1 for the left hemisphere and A2 for the right hemisphere). The recording is characterized predominantly by paroxysmal sharp-slow wave complexes, localized primarily over bilateral frontal electrodes and the right anterior temporal region. **(C)** The data filtering strategy for whole exome sequencing in this study.

The proband (II-1), a 4-year-old girl, was admitted to the hospital for evaluation of paroxysmal motor events occurring upon awakening (Figure 1A). Medication history revealed that the proband developed pallor, vomiting, and recurrent focal-to-bilateral tonic–clonic seizures beginning at 3 months of age. The seizures originated in the right hand with versive head and eye deviation to the right, subsequently propagating to the ipsilateral upper limb, lower limb, and orofacial region. Over the preceding 3 months, the patient experienced multiple seizure types: (i) focal impaired awareness seizures characterized by transient staring spells with unilateral gaze deviation and brief motor arrest lasting approximately 10 s, followed by post-ictal confusion; and (ii) focal aware motor seizures manifesting as involuntary upper-limb myoclonic jerks and epileptic drop attacks (e.g., unintentional dropping of objects). The EEG was recorded using the standard international 10–20 electrode array, mainly presenting paroxysmal sharp-slow complex waves, with the main areas being the bilateral frontal electrodes and the right temporal region (Figure 1B). Brain magnetic resonance imaging (MRI) revealed no structural abnormalities. Developmental assessment indicated a developmental quotient (DQ) of 108 and an intelligence quotient (IQ) of 105, both within the normal range. Following initiation of phenobarbital therapy, seizure frequency decreased significantly. Family history was negative for epilepsy and related neurodevelopmental disorders in both parents.

### Genetic analysis

DNA paternity testing revealed complete allelic concordance across all 15 STR loci and the sex-determining locus between the proband and both alleged parents, confirming biological parentage with a combined paternity index of 99.99%. The proband was selected as the index case for whole exome sequencing analysis. Genomic DNA was extracted from peripheral blood lymphocytes using the PicoPure™ DNA Isolation Kit, in strict accordance with the manufacturer’s protocol. Exome enrichment was performed using the Agilent SureSelect Human All Exon V6 capture system, followed by high-throughput sequencing on the Illumina HiSeq X Ten platform. The BerryGenomic Institute (Beijing, China) provided centralized whole exome sequencing services, encompassing target enrichment, massively parallel sequencing, read alignment to the human reference genome (GRCh38), variant calling, initial quality-based filtering, and functional annotation. Detailed data filtering criteria were implemented as previously reported in Figure 1C (Liu et al., 2024). Sanger sequencing validation was carried out for the identified pathogenic variant in *SETD1A*. PCR primers were designed using Primer Premier 5 software (primer sequences available upon request).

**Figure 2:**
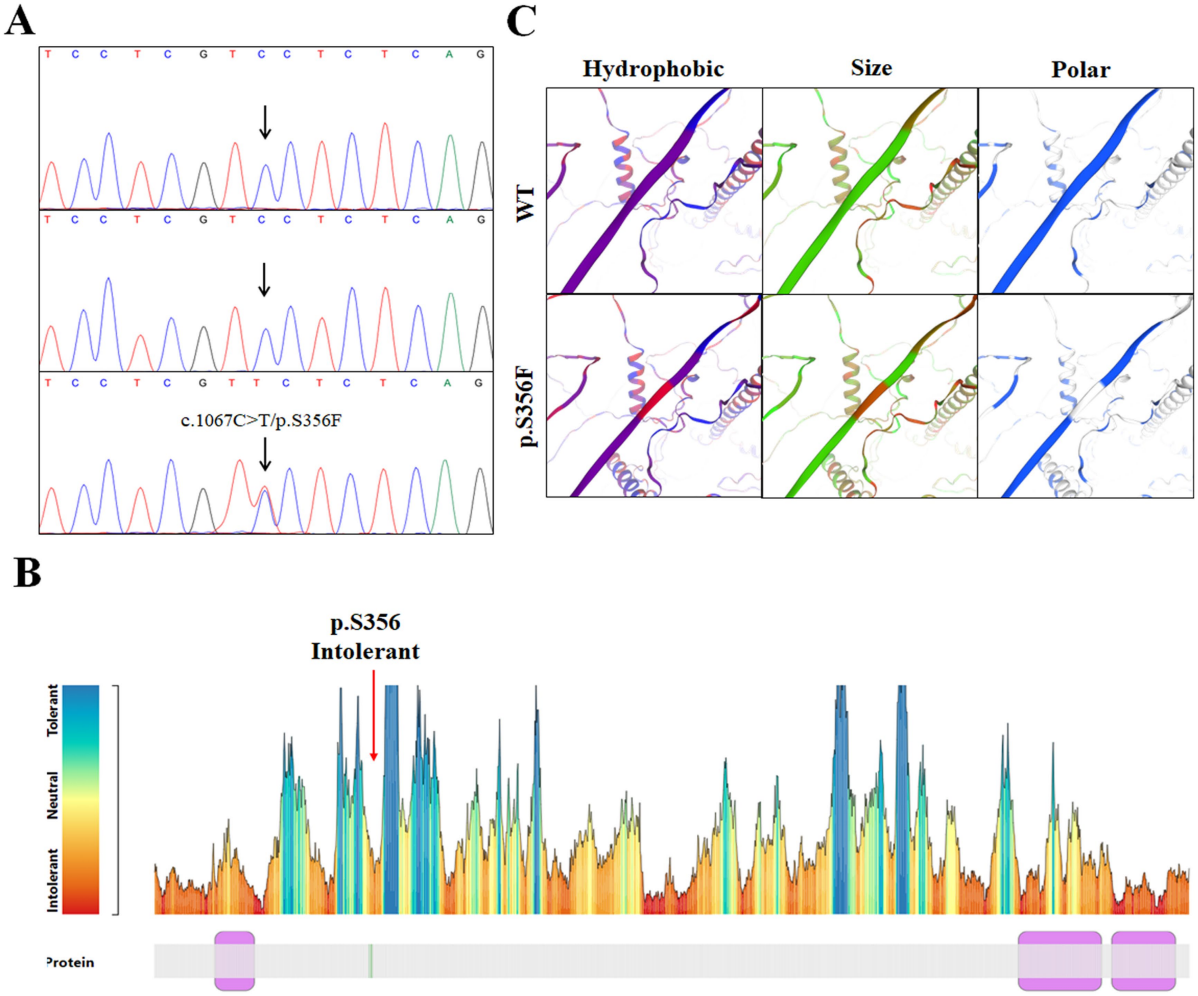
Genetic analysis of the family. **(A)** Sanger DNA sequencing chromatogram demonstrates a *de novo* heterozygous mutation (c.1067C > T/p.S356F) of *SETD1A* in the patient. **(B)** An analysis of the tolerance of the SETD1A protein’s amino acid sequence indicates that the mutation site was in an intolerant region. **(C)** Swiss-model program predicted the mutation disrupts the hydrophobic surface area, polar and size of SETD1A protein.

Whole-exome sequencing generated 9.52 Gb of high-quality data, achieving 99.6% target region coverage and 98.2% of target bases covered at a depth of ≥10×. After alignment and single nucleotide variant calling, 69,715 variants were identified in the proband. After data filtering and Sanger sequencing validation, only one novel heterozygous mutation (NM_014712.3: c.1067C > T/p. S356F) of *SETD1A* was found in the proband (Figure 1C). No other mutations that conform to the genetic pattern or associate with neurodevelopmental related phenotypes were identified (Table 1). Sanger sequencing further confirmed that this variant was a *de novo* mutation, which only exists in the proband and absence in both parents (Figure 2A). The novel mutation, resulting in a substitution of serine by phenylalanine, was absent in our 200 local control cohorts and precited to be deleterious (Table 2). MetaDome software predicted that p.S356 amino acid is in the intolerant region of SETD1A protein (Figure 2B). SWISS-MODEL online software analysis indicated that the p.S356F substitution alters the hydrophobic surface area, polar and size of SETD1A protein (Figure 2C). According to American College of Medical Genetics and Genomics and the Association (ACMG) guideline (Richards et al., 2015), the mutation met the likely pathogenic criteria (PS2 + PM2 + PP3).

**Table 1:** The gene list of Sanger sequencing validation.

Chr	Pos	RB	AB	Gene	Mutation	gnomAD	OMIM
5	141123572	C	A	PCDHB4	NM_018938.4: c.1574C > A:p.A525E	0.007246	–
5	141123589	G	A	PCDHB4	NM_018938.4: c.1591G > A:p.G531S	0.001276	–
6	39056380	A	G	GLP1R	XM_017010750.1: c.77A > G:p.Y26C	0.003201	–
7	99195315	T	C	KPNA7	XM_017012216.1: c.20A > G:p.N7S	1.98E-05	AR: embryo maturation arrest
9	138121717	G	C	CACNA1B	NM_000718.4: c.6738G > C:p.Q2246H	0.000394	AR: neurodevelopmental disorder
11	44124941	G	A	EXT2	NM_000401.3: c.995G > A:p.R332H	0.00023	AD: exostoses; AR: seizures, scoliosis, and macrocephaly syndrome
15	51503020	T	A	DMXL2	NM_001378464.1: c.2538A > T:p.E846D	1.31E-05	AD: deafness; AR: epileptic encephalopathy
16	30964809	C	T	SETD1A	NM_014712.3: c.1067C > T:p.S356F	.	AD: epilepsy
18	70169048	T	C	RTTN	NM_173630.4: c.1496A > G:p.E499G	.	AR: microcephaly, short stature, and polymicrogyria with seizures

Chr, Chromosome; Pos, position; RB, reference sequence base; AB, alternative base identified; AR, autosomal recessive; AD, autosomal dominant.

**Table 2:** The bioinformatic analysis of p.S356F in *SETD1A.*

Software	SIFT	PolyPhen-2	MetaLR	CADD	REVEL	M-CAP	1000G	gnomAD
NM_014712.3: c.1067C > T/p.S356F	Damaging	Possibly Damaging	Damaging	21.9	Uncertain (0.292)	Damaging	0	0

## Discussion

SETD1A is located on chromosome 16p11.2 and encodes a core catalytic subunit of the SET/COMPASS (Complex of Proteins Associated with Set1) complex, a mammalian H3K4 methyltransferase family member (Chen et al., 2023). Since the initial identification of SETD1A loss-of-function variants in schizophrenia patients by Takata et al. in 2014, this gene has been established as a significant risk gene for neurodevelopmental disorders (Takata et al., 2014). Singh et al. demonstrated a significant association between SETD1A variants and schizophrenia through meta-analysis of large-scale exome sequencing data (Singh et al., 2016). Notably, the clinical phenotypes associated with SETD1A variants exhibit marked heterogeneity and age-dependent expression: early-onset manifestations in infancy primarily include developmental delay, intellectual disability, and epilepsy, whereas older children and adults more commonly present with schizophrenia, obsessive-compulsive disorder, and other neuropsychiatric symptoms (Jin et al., 2023; Su et al., 2025). Here, we identified a *de novo* mutation (NM_014712.3: c.1067C > T/p.S356F) of *SETD1A* in a Chinese patient with epilepsy. Our study may expand the mutation spectrum of SETD1A-related neurodevelopmental disorders and may contribute to the genetic counseling of epilepsy.

SETD1A-mediated H3K4 methylation is essential for precise regulation of neurodevelopment (Chen et al., 2023). H3K4me3 modifications are enriched at transcription start sites and promote gene expression by recruiting transcriptional machinery and chromatin remodeling complexes (Alicea-Velazquez et al., 2016). In neurons, SETD1A regulates genes critical for synaptic function, neuronal migration, and axon guidance. Mouse model studies have demonstrated that SETD1A haploinsufficiency impairs synaptic plasticity in the prefrontal cortex, manifesting as deficits in long-term potentiation and NMDA receptor dysfunction (Chen et al., 2022; Curtis, 2022). Notably, the p.S356F mutation identified is located outside the SET domain. Previously reported pathogenic SETD1A variants are distributed across various exons but show enrichment at the 5′ end of the gene rather than within the catalytic SET domain (Singh et al., 2016).

The mutation carrier in this study presented early-onset epilepsy, but the brain structure and intelligence are normal, which was similar to one case reported by Yu et al. (2019). However, in other previously reported phenotypes associated with SETD1A-related neurodevelopmental disorders, most patients carrying *de novo SETD1A* variants had global developmental delay or intellectual disability, and 38% experiencing seizures (Singh et al., 2016). Recent evidence suggested that partial loss of SETD1A function predominantly impairs neural circuit function in the prefrontal cortex, whereas hippocampal development appears to be largely preserved (Nagahama et al., 2020). Our study supports a possible new role of non-SET domains in neurodevelopmental processes.

Previous studies have established that loss-of-function mutations in SETD1A are associated with neurodevelopmental disorders. While frameshift and nonsense mutations constitute the majority of such pathogenic variants, several missense mutations in SETD1A have also been implicated in neurodevelopmental conditions, including epilepsy and schizophrenia (Kummeling et al., 2021; Su et al., 2025). For instance, the missense variants p.S575P and p.E857Q have been reported in individuals with schizophrenia (Morikawa et al., 2022). Yu et al. identified three *de novo* missense mutations (p.Q269R, p.G1369R, and p.R1392H) in patients diagnosed with early-onset epilepsy (Yu et al., 2019). Similarly, two additional missense variants, p.Q838R and p.L863F, were detected in patients with developmental delay or intellectual disability (Faundes et al., 2018). In the present study, we report a novel de novo missense variant, p.S356F, in SETD1A in a patient with early-onset epilepsy. This patient exhibited relatively nonspecific seizure semiology, normal brain MRI findings, and preserved cognitive function, which is similar to prior cases harboring SETD1A missense variant. Whole-exome sequencing further detected heterozygous variants in other neurologically relevant genes, including *CACNA1B*, *DMXL2*, and *RTTN* (Table 1). However, existing evidence indicated that these genes were associated with autosomal recessive inheritance patterns (Wambach et al., 2018; Gorman et al., 2019; Maddirevula et al., 2019). Thus, heterozygous variants are unlikely to be causative in this context. Taken together, the *de novo* occurrence, evolutionary conservation of the affected residue, predicted deleterious impact on protein function, and phenotypic concordance with previously reported SETD1A-associated cases further support the pathogenicity of the p.S356F variant may contribute to the patient’s epilepsy.

This study further expanded the phenotypic spectrum of SETD1A-related disorders, suggesting that variants in this gene may be associated with isolated early-onset epilepsy without necessarily accompanying severe intellectual disability or developmental delay (Yu et al., 2019). This finding carries significant implications for clinical genetic counseling that *SETD1A* testing should be considered for infants with unexplained early-onset epilepsy, and long-term neuropsychiatric follow-up is crucial for carriers.

In conclusion, we employed whole exome sequencing and Sanger sequencing to identify the genetic cause of a patient with early-onset epilepsy. A de novo mutation (NM_014712.3: c.1067C > T/p.S356F) of *SETD1A* was detected and predicted to be deleterious. Our study may not only expand the spectrum of *SETD1A* mutations and contribute to the genetic diagnosis and counseling of patients with epilepsy but also provide a new case with relatively isolated early-onset epilepsy associated with *SETD1A* variant.

## Data Availability

The original contributions presented in the study are included in the article/supplementary material, further inquiries can be directed to the corresponding authors.
